# Acceptability and Usability Evaluation of Finger-Stick Whole Blood HIV Self-Test as An HIV Screening Tool Adapted to The General Public in The Central African Republic

**DOI:** 10.2174/1874613601711010101

**Published:** 2017-11-21

**Authors:** Gérard Grésenguet, Jean de Dieu Longo, Serge Tonen-Wolyec, Ralph-Sydney Mboumba Bouassa, Laurent Belec

**Affiliations:** 1 Centre National de Référence des Maladies Sexuellement Transmissibles et de la Thérapie Antirétrovirale, Bangui, Central African Republic; 2 Unité de Recherches et d’Intervention sur les Maladies Sexuellement Transmissibles et le SIDA, Département de Santé Publique, Faculté des Sciences de la Santé de Bangui, Central African Republic; 3 Faculté de Médecine *et de* Pharmacie, Université de Bunia and Université de Kisangani, Democratic Republic of Congo; 4 Laboratoire de Microbiologie, hôpital Européen Georges Pompidou, Assistance Publique-Hôpitaux de Paris, and Faculté de Médecine Paris Descartes, Université Paris Descartes, Sorbonne Paris Cité, Paris, France.

**Keywords:** Self-test, HIV, Immunochromatography, Usability, WHO recommendations, French-speaking country, Sub-Saharan Africa, Central African Republic

## Abstract

**Background:**

Opportunities for HIV testing could be enhanced by offering HIV self-testing (HIVST) in populations that fear stigma and discrimination when accessing conventional HIV counselling and testing. Field experience with HIVST was poorly reported in French-speaking African countries.

**Objective:**

To investigate the usability of HIVST in Bangui, Central African Republic.

**Methods:**

The prototype self-test Exacto^®^ Test HIV (Biosynex, Strasbourg, France) was used to assess the usability of HIVST in 300 adults living in Bangui, according to WHO technical recommendations. Simplified and easy-to-read leaflet was translated in French and Sango.

**Results:**

Preliminary survey in 3,484 adult volunteers including students, men who have sex with men and female sex workers living in Bangui showed that previous HIV testing in conventional centres for HIV counselling and testing was relatively infrequent and that acceptability of HIVST was elevated, although high heterogeneity could be observed between groups. The notice in French and Sango of Exacto^®^ Test HIV were chosen in 242/300 (80.6%) and 58/300 (19.4%), respectively. It was correctly understood in 273/300 (91.0%). The majority (275/300; 91.6%) correctly performed the HIV self-test; however, 71/300 (23.0%) asked for oral assistance. Most of the participants (273/300; 91.0%) found that performing of the self-test was very easy or easy, and less than Most of participants (273/300; 91.0%) found that performing of the self-test was very easy or easy and less than 1.0% (2/300) found it difficult. Overall the result were correctly interpreted in 96.9% (3,782/3,900), the reading/interpretion errors concerned the positive (96/1,800;5.3%), invalid (17/600;2.8%) and negative (5/1,500; 0.3%) self-test. The Cohen's coefficient κwas 0.94. The main obstacle for HIVST was the educational level, with interpretation difficulties in poorly educated people.

**Conclusions:**

Our observations on profane adults living in Central African Republic, demonstrate: (i) the need to adapt the notice of instruction to African public, including educational pictograms as well as notice in vernacular language(s); (ii) the frequent difficulties in understanding the notice with frequent misinterpretation of test results; (iii) and the generally good usability of the HIV self-test despite these latter pitfalls. More research on exploring the best strategy (*i.e*. supervised *versus* unsupervised strategies) for different high- and low- risk populations in resource-constrained settings remains needed.

## 1 INTRODUCTION

Reaching universal HIV status awareness is crucial to ensure that all HIV-infected patients will have access to antiretroviral treatment and achieve virological suppression. Diagnosing 90% of all people with HIV is the first of the three global 90–90–90 targets set by the United Nations to end the HIV epidemic by 2030 [1]. The first 90 goal of the UNAIDS initiative remains however a key challenge [2]. Indeed, despite achievements in scaling up HIV testing during the 10 last years, more than 45% of people living with HIV in sub-Saharan Africa are unaware of their HIV status [3]. Pitfalls of HIV testing in health care facilities include fear of privacy and confidentiality loss, and possible stigma and discrimination, especially in young people and marginalized high-risk groups, such as female sex workers (FSW) and men who have sex with men (MSM) [4-6].

HIV self-testing (HIVST) represents an innovative strategy to expand access to HIV testing services in the general population and also to reach individuals at high risk for HIV who may not otherwise carry out HIV testing, including young people and key populations [7, 8]. In 2012, oral HIVST was approved by the US Food and Drug Administration in the USA [9]. In 2015, HIVST was authorized in Europe [10]. In low-income countries, regulated self-test kits are generally not yet available for the general public [6]. However, recent pilot studies on the field conducted in Kenya [11, 12], Malawi [13], Nigeria [14], Uganda [15] and South Africa [16, 17] have demonstrated high acceptability and uptake of HIVST in sub-Saharan Africa [18], and have shown that interventions involving HIVST may be effective in linking self-testers to effective HIV care. However, HIVST only does not appear to increase linkage to care and studies have shown that additional interventions may be required [19]. Based on these preliminary observations, HIVST has been proposed as a way to address the challenges of efficiently reaching people at risk for or with an undiagnosed HIV infection who may not test otherwise [20]. The World Health Organization (WHO) encourages henceforth countries to pilot and explore how HIVST can be used to scale up HIV testing, especially among people who do not have access to the existing HIV testing services [6, 21]. Donors and stakeholders are also currently evaluating whether investments should be made to support the development, promotion, and marketing of HIVST in low-income countries [6, 22]. In December 2016, WHO formally recommended HIVST as an additional approach to HIV testing services [23, 24]. Finally, the WHO has published technical recommendations to develop and validate HIV self-tests [25-27].

Pilot programmes on the field will provide the experiences needed to deliver HIVST in an effective, ethical and acceptable way and to inform country implementation and policy. In Africa, the opportunities and barriers afforded by HIVST have been until now exclusively explored in English-speaking countries [6, 28-30]. To our knowledge, the acceptability, feasibility and usability of HIVST has been poorly reported in French-speaking African countries, which have their own specific cultural, economic, and societal characteristics. Finally, the aim of this study was to carry out the usability evaluation of HIVST in Bangui, Central African Republic, using a prototype HIV self-test and according to recent WHO technical recommendations [20, 25]. Thus, the usability evaluation included the 3 following substudies: i) Questionnaire to assess whether key messages and instructions from packaging and labelling are understood by untrained intended users; ii) Interpretation by untrained users of simulated rapid diagnostic tests (*i.e.* pre-made and contrived results); iii) Self-testing when assay is performed by untrained intended users [25].

## 2 MATERIAL AND METHODS

### 2.1 Prototype HIV Test for Self-Testing

The CE IVD, lateral flow, immunochromatographic HIV rapid test (*Own Brand Labeller*: Biosynex, Strasbourg, France; trademark, Exacto^®^ PRO Test HIV) [6, 26, 27], was adapted as a prototype finger-stick whole-blood HIV self-test (Exacto^®^ Test HIV, Biosynex). The test uses a combination of a specific antibody binding protein that is conjugated to colloidal gold dye particles and synthetic antigens (gp41, gp36) able to detect antibodies against HIV-1 or HIV-2 in whole-blood, serum or plasma, which are bound to the solid phase membrane. The Exacto^®^ Test HIV fulfilled the following criteria: i) Capillary blood-based test detecting early HIV infection; ii) Sterile safety lancet; iii) Simplified blood sampling system; iv) Simplified buffer delivery system; v) Specimen presence control by blood deposit assessment and migration control band; vi) Results in 10 minutes.

### 2.2 Survey on HIV Self-Testing Acceptability

A preliminary survey on the problematic of HIV testing mainly focused on HIVST was conducted using a self-administered questionnaire by the Ministry of Health and Population, Central African Republic, in students (>18 years) living in Bangui, including college or university students (n=1,782; 837 males, 945 females; mean age, 21 years; age range, 18-31), and the key group of MSM (n=396; mean age, 23 years; age range, 18-39) and (n=1,306; mean age, 23 years; age range, 18-47 years). The college or university students were recruited from 13 secondary schools and university places in Bangui, after permission. Only major students were subjected to anonymously answer the questionnaire. The MSM attended the *Centre National de Référence des Infections Sexuellement Transmissibles et de la Thérapie Antirétrovirale* for care, counselling and adapted intervention and treatment of sexually transmitted infections and possibly HIV infection, as described previously [31]. FSW were included in a descriptive, quantitative, population-based cross-sectional survey to assess the typology of female commercial sex work in Bangui, as described previously [32]. The population of FSW in Bangui is remarkably heterogeneous [32]. Thus, “official” or “professional” FSW (33%) who report themselves to have their main resources from paid sexual transactions are divided in two categories: the so-called “*pupulenge*” (14%), *i.e*. dragonflies consisting of roamers, who travel around the city to hotels and nightclubs seeking wealthy clients, and the category of “*kata*”, (19%), *i.e.* whores working in poor neighbourhoods. In addition, the “clandestine” or “nonprofessional” FSW (67%), constitute women who did not identify themselves as sex workers, reporting another activity as their main source of income or were still secondary or university students, but who nevertheless had sexual transactions during the prior three months and reported having at least two sexual partners outside their regular partner in this period.

A total of 3,484 adult volunteers were then subjected to a simple questionnaire on HIV testing and HIVST based on 6 principal questions depicted in the Table **1**.

### 2.3 Study Design of Usability Evaluation

 The usability evaluation of the Exacto^®^ Test HIV (Biosynex) is a multicenter cross-sectional study performed between June and July 2016 in Bangui, Central African Republic, consisting in face-to-face and self-administered questionnaires, according to the WHO recommendations [25].

### 2.4 Study Population for Usability

 Volunteers were included at 5 inclusion sites of voluntary and counselling testing for HIV infection as well as care of HIV-infected individuals, in 3 residential districts of the 2^nd^ (district Lakouanga and Sica), 4^th^ (district Fouh), and 6^th^ (district Fatima) arrondissements of Bangui, as well as in the 2 most important hospitals of the city (Hôpital Communautaire and Hôpital de l’Amitié). Included volunteers were asked for socio-demographic and personal findings (see Table **2**), including, sex, age, known ongoing pregnancy, sexual orientation, partnership and civil status, occupation, education level, number of sex partner in the past 6 months, past history of HIV counselling and testing, knowledge on its own HIV serostatus and antiretroviral treatment.

### 2.5 Recruitment

All participants were volunteers recruited from lay users (patients, their visitors/relatives) or health care workers (doctors, nurses, laboratory technicians) at the study sites. Inclusion of a limited number of health care workers is recommended by the WHO [25]. These participants, including health care workers, were either people seeking to know their HIV status, or people living with HIV. The inclusion criteria were: age ≥ 18 years, self-claimed ability to read the instruction notice of the HIV self-test in French or Sango, willingness to perform HIV screening and willingness to give written informed consent to participate to the study. The exclusion criteria were: age < 18 years, illiterate, and uncompliant regarding the protocol instructions. Note that all participants had no previous experience with HIVST at least the previous 24 months of enrolment.

### 2.6 Notice Design for HIVST and Translation in Vernacular Language

The original notice for professional use of the Exacto^®^ Test HIV was adapted into a simplified but comprehensive notice adapted to the African public, with inclusion of pictures showing African people carrying out the test. The simplified notice in French was further translated in Sango, which constitutes the most widely used vernacular language in the Central African Republic. Finally, the simplified notice for the HIV self-test was easy-to-read leaflet in French and Sango, in A3 format colour printing.

### 2.7 Study Outcomes

The usability evaluation was divided into 3 substudies carried out by trained healthcare professionals according to 2015 WHO recommendations [25]. A structured questionnaire was used to obtain socio-demographic data and to evaluate the participants’ understanding about the instruction notice and their opinion or level of satisfaction about the practicability of the HIV self-test.

#### 2.7.1 Substudy 1: Comprehension of Labelling

After explanations on the objectives of research work, the participants were asked to move in a private room, where the instruction notice of the Exacto^®^ Test HIV was given to the participant for reading and understanding in its preferred language (French or Sango). After reading the notice, the participant was asked to agree continuing the study by further completing a self-administered evaluation questionnaire about understanding the notice instructions. Seven questions restarting the key messages of the instruction notice with closed answers [i) True; ii) False] were asked by the observer on the followings items (*see* Table **3**): 1. Identification of each component of the kit; 2. Manipulation of blood sampling device; 3. Diluent deposit; 4. Possession of a timer; 5. Interpretation of a negative test result; 6. Diagnosis of an invalid test result; 7. Practical consequences of a positive test result.

The participants correctly answering to minimum five questions posed were considered to have correctly understood the instructions, whereas those only answering correctly to less than five questions were considered to have not understood the notice. The participants were using the same instruction notice for the rest of the study.

A satisfaction questionnaire concerning the instruction notice allows collecting information concerning the contents of the kit, the realization of the HIV self-test, the interpretation of the self-test results, the overall understanding of the instruction notice, and the usage of local language notice.

#### 2.7.2 Substudy 2: Interpretation of Results

All participants from substudy 1 were evaluated concerning the ability to read and interpret the results of the HIV self-test. In a confidential room supervised by an observer, 13 standardized tests (including 3 positive, 5 negative, 2 invalid and 3 positive with low test strip) were proposed to the participants for interpretation after successive random fate. These standardized tests were coded by numbers to know the expected results.

A satisfaction questionnaire concerning the interpretation of HIV self-test results allows to collect information concerning the reading and interpretation of a *positive* test, a *negative* test as well as an *invalid* test, and to check whether the blood deposit in the SQUARE well was correctly observed.

#### 2.7.3 Substudy 3: Observation of Manipulation

In a confidential room supervised by an observer, each participant received a box containing the Exacto^®^ Test HIV kit. Participant was then proposed to carry out by him self the HIVST. The healthcare professional observer was responsible for recording the respect or not of each step (see Table **4**). He was also responsible for recording the appeal of oral assistance (mimicking a telephone support), the failure factors and the elapsed time to perform HIVST (since opening the box to the migration stage) on a standardized sheet. The successful performing of the HIV self-test was conditioned by the presence of the control strip and the test result was read and recorded independently by both the participant and the observer. At the end of the session, the participant was asked to fill in a satisfaction questionnaire (*see* Table **5**). Finally, the participant moved to the next room with a trained staff member for the blood sampling. Note that the voluntary counselling and testing service provided pre-test and post-test counselling. It was signified to participants to consider only the results of HIV testing according to the national HIV screening algorithm because the HIV self-test should be used only for research purpose.

A satisfaction questionnaire concerning the realization of the HIV self-test allows collecting information concerning the recognition of the components of the HIV self-test, the overall realization of the self-test, and the ability to surmount the difficulties encountered.

## 2.8 Statistical Analysis

 All data were entered in an Excel file and analysed on SPSS 20.0 (Chicago, IL). Descriptive statistics were computed. Means and standard deviation (SD) were calculated for quantitative variables and proportions for categorical variables. The variable “education level” included 3 categories: low (unschooled and primary schooled), middle (high school, college or technical school) and high (undergraduate degree, graduate degree and highest degree awarded by a graduate school) education levels. The results were presented as a 95% confidence interval (CI) using the Wilson score bounds. The Pearson χ_2_ test was used for comparison of the frequencies, while the exact test of Fisher was used when the validity conditions of the later test were not verified. Comparisons of means used the Student *t* test or ANOVA for variables with more than two classes. The concordance between the results read by participants in connection with the expected results read by operator was estimated by the Cohen’s κ coefficient. The degree of agreement was sought after as ranked by Landlis and Koch [33]. Finally, to delineate and control the possible confounders within the study variables and to determine the independent predictors of the understanding of instruction notice (substudy 1), the correct interpretation of the HIV self-test results (substudy 2), the need of oral assistance (substudy 3) and the successful performing of the HIV self-test (substudy 3), multivariable logistic regression analysis was carried out using significant variables from the bivariate analysis, which were arbitrarily taken as references for analyses. All study variables including demographic characteristics and medical history (shown in the Table **2**) were taken into account of the bivariate and further multivariate analysis for all 3 substudies. Missing data in multivariate logistic regression analysis was assigned the null value for conservative estimates. The strength of statistical associations was measured by crude and adjusted Odds ratios (OR) and their 95% confidence intervals. P < 0.05 was considered as statistically significant.

## 3 RESULTS

### 3.1 Survey on HIV Testing and HIVST Acceptability

The results of the answers to the questionnaire on HIV testing and HIVST in various populations of adults living in Bangui are depicted in the Table **1**. Interestingly, previous HIV testing in conventional centres for HIV counselling and testing was relatively infrequent, especially in male students (18%), *kata* FSW (15%) as well as MSM population (9%). The majority of individuals involved in the survey had ever heard of HIVST (77-98%), were willing to use HIVST if it was available (69-97%), though that post-testing counselling is necessary to HIVST (74-95%) and were willing to test their partner using HIVST kits (52-98%). Pre-test counselling before HIVST was generally considered as unnecessary. Finally, some groups were willing to buy HIVST, if available, mainly male students (77%), MSM (92%) and *pupulengue* FSW (94%), whereas others groups, including female students (32%) and *kata* FSW (21%), did not. Finally, the acceptability of HIVST may be globally estimated as elevated, although high heterogeneity of answers could be observed between groups.

### 3.2 Study Population For Usability

 The demographic characteristics and medical past history of the study population for usability evaluation are presented in Table **2**. A total of 309 volunteers were recruited for the study, but 9 were excluded because they were minor (n=7) or considered as noncompliant (n=2). Finally, 300 participants, including 220 (73.3%) lay users [110 (36.7%) from 2^nd^ (Lakouanga and Sica), 55 (18.3%) from 4^th^ (Fouh), 55 (18.3%) from 6^th^ (Fatima) arrondissements of Bangui] and 80 (26.7%) health care professionals working in Hôpital Communautaire and Hôpital de l’Amitié, participated to the usability study. Female participants were 49.7% and included 9 pregnant women (6.0%). The majority was less than 30 years old. The majority was heterosexual (95.3%), single (47.7%) or married (44.0%). One-third were students, one-half employed and a minority unemployed. Low education level was observed in 10.3% of participants; middle level in 7.0% and good or high in 82.7%. The number of sexual partners during the last 6 months was generally from 1 to 5. Around half participants (48.0%) had yet received HIV counselling and testing and were aware of their HIV status; 7.7% were followed up at clinical centres for HIV infection of whom 43.4% (10/23) claimed they were taking antiretroviral treatment.

All participants were included in the 3 following sub-studies.

#### 3.2.1 Substudy 1

 The substudy 1 evaluated the ability of the 300 study participants to understand the instruction use of the HIVST Exacto^®^ Test HIV. The instruction notice in French (80.6%) was more used than that in vernacular language (Sango) (19.4%), as shown in Fig. (**1**). According to educational level, the notice in Sango was used in 35.5% (11/31) of participants with low education levels, 27.7% (28/101) of those with a middle education level and only 11.9% (20/168) of those with a high education level (P < 0.001).

All participants had taken sufficient time to read the instruction for use of the Exacto^®^ Test HIV. The analytical results of the evaluation questionnaire are shown in Table **3**. Overall, 273 (91.0%; 95% CI: 87.8‒94.2) participants understood correctly the instruction for use, *i.e.* answered correctly to at least five questions. Most (95.3%) participants correctly answered to the issue concerning the identification of kit components.

The health care workers showed a trend to better understand the instruction notice than the lay users, but the difference did not reach statistical significance (95.0% *versus* 89.6%; P=0.14). Instruction notice understanding was associated with the education level and language use. Thus, correct interpretation involved only 80.0% of participants with a low education level and 85.1% of those with middle education level and as high as 96.4% of those with high education level (P < 0.001). Furthermore, correct interpretation involved only 86.2% of participants using the instructions in Sango and 92.1% of those using the notice in French, but the difference did not reach statistical significance (P=0.15).

Age of participants was associated with the instruction notice understanding. Thus, 78.5% (n=113) of participants between 18 to 29 years interpreted correctly the notice, 89.4% (n=67) of those between 30 to 39 years and only 60.4% (n=49) of those having 40 years and more (P < 0.01).

In multivariate logistic regression analysis, none of the variables “instruction notice understanding”, “age”, “educational level”, “language used for the instruction notice” and “rapid test experience level” (*i.e.* unexperienced for lay users or experienced for health care worker) remained associated.

Results of the satisfaction questionnaire concerning the instruction notice are shown in Table **5**. One-third (33.3%) participants found that the instruction notice was very easy to understand and half (50.0%) rather easy, whereas 11.0% found it rather difficult to understand and 5.7% very difficult. A large majority (71.3%) of participants had a favourable opinion on the possibility to use the instruction notice in vernacular language; 47.0% of participants found the use of vernacular language useful and 24.3% essential.

#### 3.2.2 Substudy 2.

**Fig. (2):**
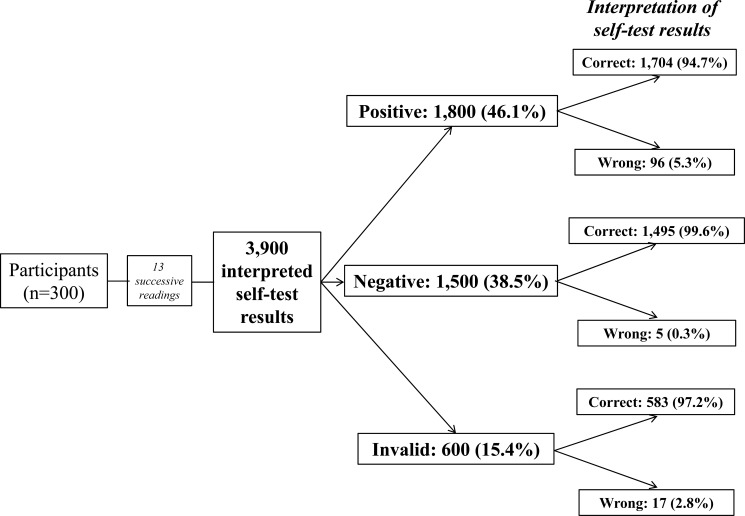
Flow chart showing the ability of participants to read and interpret (correctly or wrongly) the 3,900 results of the Exacto^®^ Test HIV (Biosynex) obtained from successive random selection of a panel of 13 standardized tests, including 6 positive, 4 negative or 2 invalid.

The substudy 2 evaluated the ability of participants to read and interpret the HIV self-test results from a panel of 13 standardized tests drawn successively. The results are depicted in the (Fig. **2**).

A total of 3, 900 standardized tests (including 1,800 positive tests, 1,500 negative tests, 600 invalid tests and 966 positive tests with low test bands) were read and interpreted by the 300 participants; 3,782 (96,9%; 95% CI: 96.4‒97.4) tests were correctly interpreted, whereas 118 (3.1%; 95% CI: 0.2‒6.2) tests were misinterpreted. Misinterpretation concerned in 5.3% positive tests (including 4.2% slightly positive which were incorrectly interpreted as “do not know” and 1.3% as invalid); in 2.8% invalid tests (including 1.8% tests which were falsely interpreted as positive and 1.0% as negative); in only 0.3% negative tests were incorrectly interpreted as “do not know”. The Cohen’s κ coefficient between the results of reading by participants and the expected results was 0.94, demonstrating excellent concordance according the Landis and Koch’s rank.

In bivariate analysis, the variables “educational level”, “language used for the instruction notice”, and “understanding of the notice” were factors associated with interpretation of HIV self-test results. In multivariate logistic regression analysis, the variable “educational level” remained associated with the interpretation of positive and invalid test results: correct interpretation of positive tests was higher in participants with high educational level than in those with middle (100.0% *versus* 95.1%, P < 0.0001; OR: 100 [95% CI: 98.8‒100.0], adjusted OR: 100 [95% CI: 98.9‒100.0]) and low (100.0% *versus* 90.6%, P < 0.0001; OR: 100 [95% CI: 98.8‒100.0], adjusted OR: 100 [95% CI: 98.9‒100.0]) educational levels; correct interpretation of invalid tests was also higher in participants with high educational level than in those with low (90.6% *versus* 84.9%, P < 0.001; OR: 100 [95% CI: 98.9‒100.0], adjusted OR: 100 [95% CI: 98.8‒100.0]) educational level.

Results of the satisfaction questionnaire concerning the interpretation of HIV self-test results are shown in Table **5**. Most (55.7%) participants found that the interpretation of positive tests was rather easy, and 33.0% very easy, whereas 11.3% participants responded that it was difficult (including 9.0% rather difficult and 2.3% very difficult). The correct observation of blood deposit in the SQUARE well of the Exacto^®^ Test HIV involved 86.6% of participants.

#### 3.2.3 Substudy 3

 The substudy 3 evaluated the ability of participants to use the finger-stick whole-blood self-test and getting a valid result in a supervised setting. The results are presented in the Table **4**.

Generally, 275 (91.6%; 95% CI: 86.8‒96.4) participants used correctly the self-test and succeeded in obtaining an interpretable result whereas only 25 (8.3%; 95% CI: 3.1‒18.5) participants failed. The successful performing of HIVST was less frequent in lay users (78.2%) than health care workers (91.2%) (P < 0.01). A total of 71 (23.0%; 95% CI: 13.3‒32.7) participants had asked for an oral assistance which 25.4% were among lay users and 18.8% among health workers (P > 0.05). Most (74.3%) of participants appealing oral assistance did it when using the lancing. The use of the lancing was the step that needed much oral assistance (91.0%). In 5 (1.6%) participants, self-testing was impossible to achieve because of the difficulty in self-pitting.

Only 19.8% of participants using the instruction notice in French received oral assistance, as compared to those using the notice in Sango (39.7%) (P < 0.01). The level of education also influenced the appeal of oral assistance: 51.6% of participants with a low level; 29.7% with a medium level; and 14.8% with a high level asked for an oral assistance (P < 0.01). Notwithstanding, the level of education had no influence on obtaining an interpretable result.

All participants (100%) with a positive self-test result read and interpreted correctly their result. Furthermore, none participant with a negative result interpreted it incorrectly as positive result. Therefore, the Cohen’s κ coefficient of the concordance between the reading of the results by participants and by operator was 1.0.

Finally, the vast majority of participants correctly recognized the blood deposit in the SQUARE well and the diluent deposit in the ROUND well of the Exacto^®^ Test HIV, in 97.3% and 97.6%, respectively (Table **4**).

In multivariate logistic regression analysis, only the variable “need of oral assistance” remained associated with the “understanding of the notice”: the need of oral assistance was more frequent when the notice was poorly understood (39.9% *versus* 15.3%, P< 0.001; OR: 0.31 [95% CI: 0.19‒0.61]). Note that the performing of HIVST was not associated with any variables, including in bivariate analysis “successful performing of HIVST”, “age”, “educational level”, “language used for the instruction notice”, “rapid test experience level”, “understanding of the notice” and “need of oral assistance”.

Results of the satisfaction questionnaire concerning the realization of the HIV self-test are shown in Table **5**. Most (92.6%) participants found that the identification of the kit components was easy (56.3% rather easy; 36.3% very easy); 57.3% of them responded that the performing of the self-test was rather easy, 33.7% very easy, 8.3% rather difficult and 1.0% very difficult. When asked about the ability to surmount the difficulties encountered during the performing of the self-test: 84.7% of participants found it easy (58.7% rather easy; 26.0% easy) and only 15.3% found it difficult (11.3% rather difficult; 4.0% very difficult).

### 4 DISCUSSION

We herein evaluated the acceptability of HIVST and usability of a prototype immunochromatographic HIV self-test in Bangui, Central African Republic, according to WHO recommendations [25].

The Central African Republic is the one of the Central African countries the most affected by generalized epidemic of HIV, with an overall prevalence in adult population of 4.9% in 2012 [34-36]. The principal mode of HIV transmission in the Central African Republic is heterosexual [36]. The HIV epidemic in the country depicts a trend towards feminization, with an HIV prevalence twice elevated among women (7.8%) than men (4.3%) in the same age group [34]. The dynamic of HIV epidemic in heterosexual population is particularly affected by the serial or concomitant occurrence of sexually transmitted infections [37-40]. Fighting the HIV epidemic in sub-Saharan Africa remains a major issue, especially in the Central African Republic, a country where HIV epidemic was qualified as “out of control” [41].

Preliminary survey in 3,484 adult volunteers including students, MSM and FSW living in Bangui showed that previous HIV testing in conventional centres for HIV counselling and testing was relatively infrequent and that high acceptability of HIVST was elevated in major high-risk groups from the Central African Republic, although high heterogeneity could be observed between groups. High acceptability of HIVST has been previously reported in sub-Saharan Africa as an acceptable HIV testing alternative for high-risk groups [18, 29, 42], including students [43], MSM [29], and FSW [29]. The fact that the majority (52-98%) of individuals involved in the survey were willing to test their partner using HIVST kits makes concern about the risk that HIVST could lead to potential coercion between partners, a possible disadvantage which should be further addressed in the Central African population.

The results of usability showed that the notice of instruction in French was preferentially used in 8 of 10 participants, and that the notice in the vernacular language Sango was also frequently preferred, in nearly 20% of participants, stressing for the first time the need for translation and adaptation of the user's guide in vernacular language(s). However, the notice was correctly understood in the vast majority (91.0%) of the volunteers. The final results were correctly interpreted in 96.9% of the cases, with reading or interpretation errors for 5.3% of the positive results, 2.8% of the invalid results and 0.3% of the negative results. Finally, the HIV self-test was correctly carried out in the majority of volunteers (91.6%) of cases, although oral assistance was required in 23.0% of cases. In a minority of participants (1.6%), self-testing was difficult or impossible to achieve because of the difficulty in self-pitting. In the present series, the lower was the educational level of profanes, the greater was the difficulty in performing the HIV self-test and to interpret it correctly. These observations, novel in French-speaking sub-Saharan Africa, on a large number of lay adults living in Central Africa, demonstrate: i) the need to adapt the notice of instruction to African public, including educational pictograms as well as notice in vernacular language(s) in addition to French language; ii) the frequent difficulties in understanding the notice of instruction in addition to frequent misinterpretation of test results; iii) and the generally good usability of the HIV self-test despite these latter pitfalls. Taken together, the study highlights the feasibility of HIVST in French-speaking settings, but emphasizes the need to likely control the availability of HIVST in poorly educated people.

### 4.1 Adaptation of The Notice of The Exacto^®^ Test HIV to The African Public

 For HIVST evaluation, the WHO recommends that the participants would be expected to be representative of intended users of the device and include those with different levels of education and socioeconomic status [25]. Specifically, we first took into consideration that a significant proportion of population living in French-speaking sub-Saharan Africa, especially in Central Africa, is uneducated or poorly educated, and thus uses local vernacular language(s) instead of French language [44].Therefore, the translation of instruction notice of the HIV self-test in vernacular language appeared an important pre-requisite for the study. In practice, the simplified French notice was translated in Sango, which constitutes the most spoken national vernacular language of the Central African Republic [45]. Note that Sango language is not commonly taught at schools in the Central African Republic, because it is spoken universally by the whole population.

### 4.2 Usability of the Exacto^®^ Test HIV with Notice Adapted to The General African Public

 The usability evaluation was carried out according to the WHO recommendations [25].

#### 4.2.1 Substudy 1.

 The substudy 1 evaluated the ability of the study participants to understand the instruction use of the Exacto^®^ Test HIV. Overall, the vast majority (91.0%) of participants who were not previously trained, correctly used the finger-stick whole blood self-test either autonomously or with oral assistance, and thus understood correctly the instruction for use. The vernacular language Sango was frequently used by poorly and middle educated people, but the final understanding of the instruction notice appeared relatively independent of the educational level as well as the use of French or vernacular languages. These observations demonstrate the importance of the translation of instruction notice into vernacular reference to increase public accessibility to HIVST, but also point the difficulties of such translation to provide easy reading and understanding in poorly educated users.

Most participants found that the written and visual information was easy to read, interpret and execute. These findings clearly validate the use of the Exacto^®^ Test HIV by the general public living in Bangui, underline the interest to use an instruction notice in the vernacular language Sango in lay users, and point the recurrent problem of low educational level as possible obstacle to correctly carry out HIVST.

#### 4.2.2 Substudy 2.

 The substudy 2 allowed evaluating the ability of participants to read and interpret the Exacto^®^ Test HIV results. Most (88.7%) participants claimed that the interpretation was rather easy or very easy. Furthermore, from a panel of 13 standardized tests drawn successively, the vast majority (96.9%) were correctly interpreted, whereas a minority (3.1%) were misinterpreted. The frequency of misinterpretation in our study population is reminiscent to that reported by Prazuck and colleagues in a non-trained general population living in France in which the majority (97.1%) of participants succeeded, with only 2.9% of the participants making errors [10]. Whatever, the instruction for use on the Exacto^®^ Test HIV packaging clearly alerts persons that a very recent risk of HIV infection (within the last three months) may result in a false negative and an additional test should be performed after the 3-month window period.

#### 4.2.3 Substudy 3.

The substudy 3 evaluated the ability of participants to use the Exacto^®^ finger-stick whole-blood self-test and get a valid result in a supervised setting. The vast majority (91.6%) of participants used correctly the self-test and succeeded in obtaining an interpretable result whereas less than 1 of 12 (8.3%) participants failed. The correct use of the lancing to collect capillary blood was the most difficult step encountered, that was the first concern raised in oral assistance (64.2%). A total of 23.0% of participants needed technical oral assistance and support, especially those using the instruction notice in Sango, as well as those with low (34.0%) and middle (21.8%) educational level. These features emphasize the importance, and probably the need, to propose a hotline at the time of commercialization of HIV self-test. Notwithstanding, the level of education had no influence on obtaining interpretable results. All participants with a positive self-test result read and interpreted correctly their results. Furthermore, none participant with a negative result interpreted it incorrectly as positive. These findings validate the possibility of practical use of the self-test by the majority of the non-trained, general public living in Bangui.

### 4.3 Conclusion and Future Perspectives

 HIVST in French-speaking sub-Saharan Africa countries, particularly in Central African countries, was clearly poorly established. Our field experience with the self-test Exacto^®^ Test HIV demonstrates in the cultural context of Central Africa satisfactory success rates of interpretation performances and its potential for use by the general public with sufficient educational level. Our pilot study generated evidence on feasibility of operationalization of an unsupervised self-testing strategy in participants with sufficient educational level, living in the cultural context of Central Africa. Similar observations were reported from preliminary study in the Democratic Republic of Congo [46]. This successful conduct of self-tests may have been related to the use of adapted instructional notice, with adequate pictures, generally in French language and also in the national language Sango, provided to sufficiently literate participants who could comprehend them, as well as to the fact that, by accepting to participate in the study, these participants showed interest in conducting self-tests by themselves. Finally, the main obstacle for HIVST was clearly the educational level, with execution and interpretation difficulties in poorly educated people.

Two self-testing strategies, supervised and unsupervised, have been documented and evaluated globally [16, 47-52]. Our observations likely indicate the possible interest of supervised self-testing strategy in poorly educated people, in which testing and counselling processes are aided at all times by a healthcare or nonhealth professional as a counsellor to read the test results and provide counselling. Individuals seeking rapid HIV testing can perform a finger stick blood test under the direction of a counsellor trained in guiding the process and supported by good instructions developed *ad hoc* for this purpose [51], with very high rate of acceptability, feasibility and accuracy correct interpretation of test results [16]. The evidence of high acceptability for supervised strategies is clear, especially in resource constrained settings [16]. Furthermore, in supervised self-testing strategy, HIV self-test can be provided in bulk by the healthcare or community personals, thus decreasing the cost of HIVST. For poor and less literate populations who cannot afford HIV self-test or cannot comprehend easily the process of testing, as in around one of five of our study participant population, the supervised self-testing strategy may likely remain the best option.

In conclusion, our observations on profane adults living in Central Africa, demonstrate: i) the need to adapt the notice of instruction to African public, including educational pictograms as well as notice in vernacular language(s); ii) the frequent difficulties in understanding the notice with frequent misinterpretation of test results; iii) and the generally good usability of the HIV self-test despite these latter pitfalls. More research on exploring the best strategy (*i.e.* supervised *versus* unsupervised strategies) for different high- and low- risk populations in resource-constrained settings remains needed in the cultural context of Central Africa.

## Figures and Tables

**Fig. (1):**
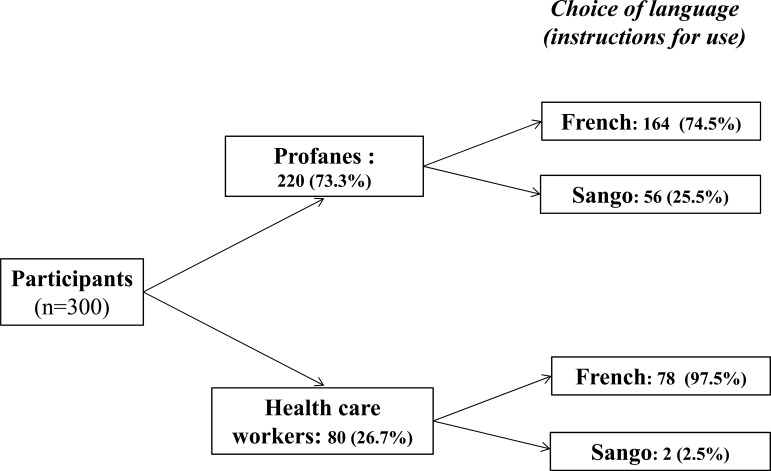
Flow chart showing the choice of language (French or Sango) of the instruction notice. The majority of participants were profanes. A subgroup of health care workers (26.7%) was also included, as requested by WHO recommendations [25].

**Table 1:** Acceptability of HIV self-test in college and university students population and key populations including men who have sex with men and professional and clandestine female sex workers living in Bangui (n = 3,484). The results are shown as number and percentage in brackets.

	**Adult student population^µ^** (n = 1,782)			**Men who have sex with men** **^µµ^** (n = 396)	**Female sex workers^µµµ^** (n = 1,306)			
					**Official** (n = 881)			**Clandestine** (n = 425)	
	**Female** (n = 945)	**Male** (n = 837)	*P* ^£^ *between female and male*		***Pupulengue*** (n = 487)	***Kata*** (n = 394)	*P* ***^£^*** *between pupu-lengue and kata*		*P* ***^£^*** *between official and clandestine* *FSW*
Ever tested for HIV in VCT *(conventional centres for HIV counselling and testing)*	236 (25)^$^	150 (18)	0.001	36 (9)	205 (42)	59 (15)	< 0.001	102 (24)	NS
Ever heard of HIVST before	926 (98)	728 (87)	< 0.001	305 (77)	414 (85)	394 (100)	< 0.001	378 (89)	NS
Willing to use HIVST if it was available	652 (69)	652 (78)	0.001	384 (97)	463 (95)	335 (85)	< 0.001	315 (74)	< 0.001
Pre-test counselling is necessary to HIVST	510 (54)	226 (27)	< 0.001	59 (15)	122 (25)	91 (23)	NS	208 (49)	< 0.001
Post-test counselling is necessary to HIVST	699 (74)	795 (95)	< 0.001	348 (88)	472 (97)	292 (74)	< 0.001	378 (89)	NS
Willing to buy HIVST	302 (32)	644 (77)	< 0.001	364 (92)	458 (94)	83 (21)	< 0.001	208 (49)	< 0.001
Willing to test partner using to HIVST kits	491 (52)	652 (78)	< 0.001	384 (97)	477 (98)	256 (65)	< 0.001	230 (54)	< 0.001

^$^Number (%); ^£^ Statistical analysis were carried out using Pearson’s χ2 test. **^µ^**The students were recruited from recruited from 13 secondary schools and university places in Bangui; **^µµ^**MSM were included at the *Centre National de Référence des Infections Sexuellement Transmissibles et de la Thérapie Antirétrovirale* for care, counselling and adapted intervention and treatment of sexually transmitted infections and possibly HIV infection, as described previously [31]; ^µµµ^FSW were included in a descriptive, quantitative, population-based cross-sectional survey to assess the typology of female commercial sex work in Bangui, as described previously [32]. Female sex worker: FSW; HIVST: HIV self-testing; MSM: Men who have sex with men; NS: Not significant

**Table 2:** The demographic characteristics and medical history of the 300 study participants.

**Variable**	**Items**	**Number (%)**
**Sex**		
	Male	151 (50.3)
	Female*	149 (49.7)
**Age**		
	18 - 29 years	144 (48.0)
	30 - 39 years	75 (25.0)
	≥ 40 years	81 (27.0)
**Sexual orientation**		
	Heterosexual	286 (95.3)
	MSM or bisexual	14 (4.7)
**Partnership and civil status**		
	Single	143 (47.7)
	Married/partnered	132 (44.0)
	Separated or divorced	13 (4.3)
	Widowed	12 (4.0)
**Occupation**		
	Student	101 (33.7)
	Employed	157 (52.3)
	Unemployed	42 (14.0)
Educational level **		
	Unschooled***	31 (10.3)
	Primary school***	21 (7.0)
	High school, college or technical school	80 (26.7)
	Undergraduate degree	107 (35.7)
	Graduate degree	28 (9.3)
	Highest degree awarded by a graduate school	33 (11.0)
**Number of sex partner in the past 6 months**		
	0	54 (18.0)
	1 ‒ 5	237 (79.0)
	6 ‒10	8 (2.7)
	> 10	1 (0.3)
**Previous history of HIV counselling and testing**		
	Yes	144 (48.0)
	No	156 (52.0)
**Attending care centres for HIV infection**		
	Yes	23 (7.7)
	No	277 (92.3)
**Taking antiretroviral treatment when followed up for HIV infection**		
	Yes	10 (43.4)
	No	13 (56.6)

* Females were pregnant in 6.0% (n=9). ** Educational level was assessed as follows: i) Unschooled; ii) Poorly educated people, *i.e.* attending primary school only; iii) Educated people, *i.e.* attending high school, college or technical school; iv) Highly educated people, with undergraduate or graduate degree; *** Unschooled volunteers and those receiving only primary education included individuals with low level of reading. MSM: Men who have sex with men.

**Table 3:** Analytical results of the evaluation questionnaire concerning the ability of the 300 study participants to understand the instruction for use of the HIVST Exacto^®^ Test HIV (Biosynex). The questions raising specific issues concerning the manipulation of the kit, the interpretation of a test result and the consequence of the test results, were asked by the observer and the answers were closed.

**Issue raised by each question (Q)**	**Answers**	**Number (%)**
→ **Q1:** “A capital letter is associated with each component of the kit to better identify it during the performing of self-test”	o True^#^	286 (95.3)
	o False	1 (0.3)
	o Do not know	13 (4.4)
→ **Q2:** “The blood collection device helps to collect the blood and place it immediately into the SQUARE well of self-test”	o True^#^	282 (94.0)
	o False	5 (1.7)
	o Do not know	13 (4.3)
→ **Q3:** “Two drops of diluent should be placed in the same well as the drop of blood”	o True	23 (7.7)
	o False^#^	259 (86.3)
	o Do not know	18 (6.0)
→ **Q4:** “A timer (watch or mobile) to clock 10 minutes before reading the result is needed”	o True^#^	232 (77.3)
	o False	15 (5.0)
	o Do not know	53 (17.7)
→ **Q5:** “Lack of band by test result is interpreted as a negative test”	o True	39 (13.0)
	o False^#^	164 (54.7)
	o Do not know	97 (32.3)
→ **Q6:** “Lack of control band by test result should interpreted as an invalid test”	o True^#^	178 (59.3)
	o False	53 (17.7)
	o Do not know	69 (23.0)
→ **Q7:** “In case of positive self-test result, a doctor should be consulted to confirm the result”	o True^#^	226 (75.3)
	o False	13 (4.3)
	o Do not know	61 (20.4)

^#^Correct answers

**Table 4:** Analytical results of the manipulation observation concerning the ability of the 300 study participants to use correctly each step of the self-test Exacto^®^ Test HIV (Biosynex) manipulation autonomously or with oral assistance.

**Issue raised by each questions (Q)**	**Items**	**Number (%)**
→**Q1**: “The participant recognized the different components of the kit”	o Yes	286 (95.3)
	o No	13 (4.3)
	o Oral assistance	1 (0.4)
→**Q2**: “The participant washed his hands”	o Yes	285 (95.0)
	o No	14 (4.6)
	o Oral assistance	1 (0.4)
→**Q3**: “The participant found the self-test in the bag”	o Yes	291 (97.0)
	o No	9 (3.0)
	o Oral assistance	0 (0.0)
→**Q4**: “The participant opened the diluent vial”	o Yes	290 (96.7)
	o No	10 (3.3)
	o Oral assistance	0 (0.0)
→**Q5**: “The participant disinfected his chosen fingertip with the disinfectant wipe”	o Yes	283 (94.3)
	o No	15 (5.0)
	o Oral assistance	2 (0.7)
→**Q6**: “The participant wiped residual alcohol with the compress”	o Yes	267 (89.0)
	o No	30 (10.0)
	o Oral assistance	3 (1.0)
→**Q7**: “The participant applied the lancing on the chosen fingertip and push the other tip to sting”	o Yes	220 (83.3)
	o No	60 (10.0)
	o Oral assistance	20 (6.6)
→**Q8**: “The participant pressed gently on the fingertip to obtain a drop of blood”	o Yes	280 (93.3)
	o No	14 (4.7)
	o Oral assistance	6 (2.0)
→**Q9**: “The participant placed in contact the drop of blood with the sampler tip until the tip is full”	o Yes	288 (96.0)
	o No	6 (2.0)
	o Oral assistance	6 (2.0)
→**Q10**: “The participant checked that the sampler tip is filled with blood”	o Yes	292 (97.3)
	o No	8 (2.7)
	o Oral assistance	0 (0.0)
→**Q11**: “The participant placed the blood into the SQUARE well BLOOD of the test cassette”	o Yes	292 (97.3)
	o No	6 (2.0)
	o Oral assistance	2 (0.7)
→**Q12**: “The participant shed two drops of diluent in the ROUND well DILUENT of the test cassette”	o Yes	293 (97.6)
	o No	6 (2.0)
	o Oral assistance	1 (0.4)

**Table 5:** Items and results of the satisfaction questionnaire concerning the instruction notice (substudy 1), the interpretation of HIV self-test results (substudy 2) and the realization of the HIV self-test (substudy 3).

**Satisfaction Questionnaire**	**Number (%)**
→ **Instruction notice (substudy 1)**	
• Information regarding the contents of the kit	
o Sufficient	250 (83.3)
o Insufficient	34 (11.3)
o Not read	16 (5.4)
• Information concerning the realization of the HIV self-test	
o Sufficient	247 (82.3)
o Insufficient	39 (13.0)
o Not read	14 (4.7)
• Information concerning the interpretation of the HIV self-test results	
o Sufficient	219 (73.0)
o Insufficient	61 (20.3)
o Not read	20 (6.7)
• Overall understanding of the instruction notice	
o Very easy	100 (33.3)
o Rather easy	150 (50.0)
o Rather difficult	33 (11.0)
o Very difficult	17 (5.7)
• The use of local language notice	
o Essential	73 (24.3)
o Useful	141 (47.0)
o Rather useful	54 (18.0)
o Unuseful	32 (10.7)
→ **Interpretation of HIV self-test results (substudy 2)**	
• Reading and interpretation of a ***positive*** test	
o Very easy	99 (33.0)
o Rather easy	167 (55.7)
o Rather difficult	27 (9.0)
o Very difficult	7 (2.3)
• Reading and interpretation of a ***negative*** test	
o Very easy	91 (30.3)
o Rather easy	174 (58.0)
o Rather difficult	25 (8.3)
o Very difficult	10 (3.3)
• Reading and interpretation of an ***invalid*** test	
o Very easy	90 (30.0)
o Rather easy	169 (56.3)
o Rather difficult	35 (11.7)
o Very difficult	6 (2.0)
• Correct observation of blood deposit in the SQUARE well	
o Very easy	94 (31.3)
o Rather easy	166 (55.3)
o Rather difficult	31 (10.3)
o Very difficult	9 (3.0)
→ **Realization of the HIV self-test (substudy 3)**	
• Recognition of the components of the HIV self-test	
o Very easy	109 (36.3)
o Rather easy	169 (56.3)
o Rather difficult	18 (6.0)
o Very difficult	4 (1.3)
• Overall realization of the HIV self-test	
o Very easy	101 (33.7)
o Rather easy	172 (57.3)
o Rather difficult	25 (8.3)
o Very difficult	2 (0.7)
→ Ability to surmount the difficulties encountered	
o Very easy	78 (26.0)
o Rather easy	176 (58.7)
o Rather difficult	34 (11.3)
o Very difficult	12 (4.0)
